# Léiomyosarcome utérin de 7 kilogrammes

**DOI:** 10.11604/pamj.2014.18.134.4364

**Published:** 2014-06-11

**Authors:** Mehdi Kehila, Karima Makni

**Affiliations:** 1 Faculté de Médecine de Tunis, Service C du Centre de Maternité et de Néonatologie de Tunis, Tunisie

**Keywords:** Léiomyosarcome, utérus, sarcome, leiomyosarcoma, uterus, sarcoma

## Image en medicine

Nous illustrons à travers ce cas l'agressivité des sarcomes utérins et la difficulté de leur prise charge aux stades avancés. Nous rapportons le cas d'une patiente de 58 ans, qui a consulté pour une masse abdomino-pelvienne apparue depuis trois mois et augmentant rapidement de volume. L'examen a trouvé une patiente cachectique, une énorme masse abdomino-pelvienne dure, fixe, mal limitée, arrivant à 7 cm au dessus de l'ombilic. Une échographie et un scanner abdomino-pelvien ont objectivé une volumineuse masse développée au dépens de l'utérus, faisant 29*16 cm (a, b) avec dilatation pyélo-calicielle. Vu l’âge, le fibrome était peu probable et un sarcome utérin a été suspecté. Après réanimation, la patiente a eu une laparotomie médiane xypho-pubienne révélant une énorme tumeur lisse polylobée de couleur chamois, richement vascularisée (c, d), fixée. Une tentative de libération s'est soldée d'une hémorragie cataclysmique nécessitant de la mise sous noradrénaline et des transfusions sanguines. Une exérèse rapide de la tumeur a été réalisée avec une hystérectomie sub-totale compliquées d'une plaie du sigmoïde. On a complété par une intervention de Hartman. Il persistait du résidu tumoral accolé aux anses grêles et au rétropéritoine avec un saignement diffus. Un paking a été réalisé, enlevé le lendemain avec bonne hémostase. La patiente n'a jamais pu être extubée et est malheureusement décédée en réanimation après 15 jours dans un tableau de défaillance multi-viscérale. L'examen histologique a conclu à un léiomyosarcome utérin de 7 kg infiltrant le tissu adipeux et le colon sigmoïde.

**Figure 1:**
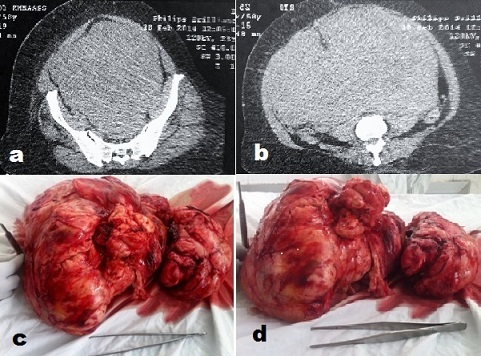
(a, b): Une échographie et un scanner abdomino-pelvien ont objectivé une volumineuse masse développée au dépens de l'utérus, faisant 29 x 16 cm avec dilatation pyélo-calicielle; (c, d): Après réanimation, la patiente a eu une laparotomie médiane xypho-pubienne révélant une énorme tumeur lisse polylobée de couleur chamois, richement vascularisée fixée

